# Synchronous appendiceal and intramucosal gastric signet ring cell carcinomas in an individual with CDH1-associated hereditary diffuse gastric carcinoma: a case report of a novel association and review of the literature

**DOI:** 10.1186/1471-230X-13-114

**Published:** 2013-07-12

**Authors:** Leslie E Hamilton, Kirsten Jones, Neal Church, Shaun Medlicott

**Affiliations:** 1 Department of Pathology and Laboratory Medicine, University of Calgary, Calgary, AB, Canada; 2 Departments of Surgery, University of Calgary, Calgary, AB, Canada

**Keywords:** Hereditary diffuse gastric carcinoma, CDH1, Signet ring cell carcinoma, Appendix, Familial cancer

## Abstract

**Background:**

Hereditary diffuse gastric carcinoma is an autosomal dominant cancer syndrome associated with mutations of the E-cadherin gene (CDH1). E-cadherin is normally involved in cell-cell adhesion, so it not surprising that individuals with this syndrome are predisposed to develop malignancies with dyshesive morphologies at a young age, such as diffuse (signet ring cell) gastric carcinoma and lobular breast carcinoma. Herein we describe the first reported case of primary appendiceal signet ring cell carcinoma arising in a CDH1-associated hereditary diffuse gastric carcinoma kindred with synchronous primary diffuse gastric carcinoma.

**Case presentation:**

A 51- year old woman, with known CDH1 mutation carrier status and a prior history of lobular breast carcinoma underwent prophylactic total gastrectomy which revealed multifocal intramucosal signet ring cell carcinoma. An appendectomy was performed at the same time due to a prior episode of presumed appendicitis, with pathologic examination significant for a primary signet ring cell carcinoma of the appendix.

**Conclusion:**

As appendiceal signet ring cell carcinoma is exceedingly rare, the occurrence of this neoplasm in this patient, with this particular morphology, provides credence for it being part of the hereditary diffuse gastric carcinoma spectrum of malignancies.

## Background

Hereditary diffuse gastric carcinoma is an autosomal dominant cancer syndrome accounting for approximately 1 to 3% of all gastric carcinoma, associated with mutations of the E-cadherin (Cadherin-1; CDH1) gene [1-3]. It is characterized by high penetrance with an approximate 80% lifetime risk of developing diffuse gastric carcinoma [2]. Early onset diffuse gastric carcinoma is pathognomonic for HDGC and the natural history is the insidious development of intramucosal signet ring cell carcinoma in early adulthood progressing to advanced gastric carcinoma by mid-adulthood, at a mean age of 40 years [1]. Female kindreds also have a predisposition for lobular carcinoma of the breast. Established clinical diagnostic criteria for HDGC, as defined by the International Gastric Cancer Linkage Consortium, include: (1) two or more first or second degree relatives with confirmed diffuse (signet ring cell) gastric carcinoma, with at least one diagnosed before 50 years of age; or (2) three or more confirmed cases of diffuse gastric carcinoma amongst first and second degree relatives, independent of age of onset [4]. Additional clinical indications for genetic testing for CDH1 mutations include: HDGC in two relatives with histologic confirmation of tumor in only one individual, diffuse gastric carcinoma in an individual < 40 years regardless of family history, or individuals and families with both signet ring cell gastric carcinoma (including one case before 50 years of age) and lobular breast carcinoma [2].

Multifocal intramucosal carcinoma of stomach is apparent by the age of 20-to-30 years, preferentially at the body-antrum interface [1]. The cumulative risk of advanced carcinoma is 1% at age 20, 4% at age 30, 21% (male) to 46% (female) at age 50 and 67% (male) to 83% (female) at age 80 [1,2,5]. Therefore management includes surveillance endoscopy (every 6 to 12 months) as early as age 16 and consideration of prophylactic gastrectomy in early adulthood. Unfortunately, the insidious and multifocal nature of early signet ring cell carcinoma diminishes the sensitivity of endoscopic detection with current techniques, and prophylactic gastrectomy should be considered for any individual with a CDH1 mutation, regardless of the results of endoscopic surveillance [1,2,6]. This is further supported by pathologic reviews of prophylactic gastrectomies in CDH1 individuals. In the largest study to date, 96% of gastrectomy specimens contained early diffuse gastric carcinoma, of which only 9% had similar lesions identified on pre-operative endoscopic biopsies. When this data was included with all previous published data on prophylactic total gastrectomies (a total of 93 cases) 92.5% of specimens contained signet ring cell lesions [7,8].

Female carriers of the CDH1 mutation also have a predisposition for lobular breast carcinoma. It is likely that there are interfamilial differences, but the cumulative lifetime risk of breast cancer is reportedly as high as 39 to 52% in HDGC kindreds [5,9,10]. Female carriers should be referred to a high-risk breast cancer screening clinic prior to the age of 40 years. Nevertheless, the penetrance for gastric carcinoma is much higher and female carriers are five times more likely to develop gastric carcinoma than lobular breast carcinoma [5]. Interestingly, female carriers have a much higher cumulative risk for gastric adenocarcinoma than male carriers in the same age cohorts [1,2,5]. Furthermore, pathologic analysis of prophylactic gastrectomies in a case series of CDH1-associated HDGC families documented a trend towards more severe disease burden in females within one cohort, with larger invasive foci and an increased total number of foci [11]. The reasoning for this is not clear; hormonal or other environmental factors presumably participate in the pathogenesis of HDGC tumors.

With respect to the molecular pathogenesis of HDGC, it is not surprising that the CDH1 mutation predisposes to malignancies with a dyshesive signet ring cell morphology such as diffuse gastric carcinoma and lobular breast carcinoma. Approximately 25-50% of families with HDGC syndrome have an inactivating germline mutation of the tumor suppressor gene Cadherin-1 (CDH1) that encodes E-cadherin [1,2,12]. E-cadherin has an important role in cell adhesion, motility, proliferation and differentiation. Its primary role is that of an epithelial transmembrane glycoprotein that functions as a cell-cell adhesion molecule; normal E-cadherin also interacts with β-catenin to form complexes with actin, mediating the linkage of cells to the cytoskeleton. When CDH1 is mutated, there is loss of normal cell adhesion and cell polarity; disaggregation of cells also promotes local invasion and metastases of neoplastic cells, and loss of CDH1 is a common molecular event in advanced sporadic malignancies [13-16]. The majority of inactivating germline mutations in HDGC families are of the truncating type (75% of cases); the remaining 25% are of the missense type [1]. Somatic inactivation of the second allele is thought to result from promoter hypermethylation, mutations of CDH1, or loss of heterozygosity [2,5,17]. Although typically there is an absence or reduced staining of neoplastic cells by E-cadherin, some studies have documented limited E-cadherin staining by immunohistochemistry in HDGC-associated carcinomas [5]. Interestingly, one genetic study of HDGC kindreds found one family with an intact second CDH-1 allele and detectable E-cadherin protein in neoplastic cells, and postulated that a 50% reduction in CDH-1 function may be enough to promote tumorigenesis; other explanations include: a dominant-negative effect of the germline mutation or a missense mutation of the wild-type allele [13]. Furthermore, 50% of sporadic cases of diffuse gastric carcinoma and lobular breast carcinoma are also associated with inactivation of CDH1 and loss of E-cadherin expression, most frequently through promoter methylation [17]; this event appears to be critical for the development of tumors with this unique morphologic phenotype.

Anecdotal occurrences of primary malignancies of the colon, lung, prostate, salivary gland and pancreas have been described in CDH-1 associated HDGC families, with rare examples of the signet ring cell variety; none of which occur at a frequency greater than the general population [18]. Colorectal carcinoma has been deemed by some to be an occasional member of the HDGC disease spectrum, with the recommendation that surveillance colonoscopy should commence in this population at age 40, or 10 years younger than the youngest diagnosis of colon carcinoma [1,2,19,20]. In the general population, colorectal carcinoma is thought to be familial in 20% of cases. Several studies have documented mutations of CDH1 in cell lines and tissue samples from colorectal carcinoma, albeit rarely [14,15,21-24]. Only three of these studies correlated the expression of E-cadherin with the morphology of the carcinoma from which the cell line/tissue sample was derived; as expected, loss of expression of E-cadherin typically correlated with a poorly differentiated/signet ring cell growth pattern [15,21,23]. Alteration of the association between E-cadherin and β-catenin may represent an alternative tumorigenesis pathway to inactivation of the APC gene in the HDGC cohort [21].

In the English literature, there are at least twenty-six reported cases of colorectal carcinoma occurring in CDH1-associated HDGC kindreds; an additional eight cases of colorectal carcinoma exist in families with a history of diffuse gastric carcinoma that have tested negative for CDH1 mutations and did not meet the criteria for hereditary nonpolyposis colon cancer (HNPCC) [8-10,12,13,15,18,24-30]. It is likely that a proportion of these cases represent sporadic colorectal carcinomas unrelated to HDGC. Albeit rare (the reported incidence is less than 1% in most case series [31-36]), signet ring cell carcinoma of the colorectum would be expected to occur more frequently in individuals with HDGC than the general population given its morphology. However there is a paucity of data on the histology of colorectal carcinomas occurring in the HDGC population, as most studies simply comment on the organ of involvement of extra-gastric malignancies. Of reported cases, only one has been diagnosed as a signet ring cell carcinoma; there is at least one additional case of a colonic signet ring cell carcinoma occurring in a family with familial gastric carcinoma that tested negative for the CDH1 mutation (and negative for HNPCC) [12,28].

Notably there is an absence of any reported cases of primary appendiceal carcinomas in HDGC kindreds in the English literature. Herein we describe a case of primary appendiceal signet ring cell carcinoma arising in a 51 year old female from a CDH1-associated HDGC kindred who underwent an interval appendectomy at the time of a prophylactic gastrectomy. This is the first reported case of synchronous gastric and appendiceal signet ring cell carcinomas in HDGC. As appendiceal signet ring cell carcinoma is exceedingly rare, the occurrence of this neoplasm in this patient provides credence for it being part of the HDGC spectrum of malignancies.

## Case presentation

A 51 year old female from a known HDGC kindred was status/post mastectomy and adjuvant chemoradiation for invasive mammary carcinoma six years prior. Her family history was significant for a sister with metastatic gastric adenocarcinoma (deceased at age 41), two aunts and one cousin with gastric carcinoma (diagnosed in 40s, two of which are deceased), and a brother and niece identified as CDH1 mutation carriers. Genetic screening, performed one year after her mastectomy, confirmed that she was also a CDH1 gene mutation carrier. She elected to undergo a prophylactic total gastrectomy; prior to this her only gastrointestinal symptom was non-specific mild constant epigastric discomfort, and two surveillance endoscopic biopsies of the stomach, one year apart, were negative for carcinoma. One month prior to surgery, she was treated medically with antibiotics at an outside community hospital for presumed acute appendicitis, a diagnosis supported by CT-imaging findings. Given this history, an interval appendectomy was completed at the time of the total gastrectomy with Roux-en-Y esophagojejunostomy. At laparotomy, the 17 x 11 centimetre-stomach lacked lymphadenopathy, and its serosa, wall and mucosa were all intact and unremarkable. The appendix was enlarged and indurated with serosal adhesions.

At gross examination, the stomach was unremarkable; representative sections were submitted for histologic examination, including ten sections each from the proximal body, mid-body, angularis (body/antrum interface), and antrum, and five sections each from the proximal and distal margins; each section was sampled to ensure circumferential representation. Ten lymph nodes were identified within the regional adipose tissue. The appendix was intact with adherent hemorrhagic adhesions of its serosa, and with clear gelatinous luminal contents; the appendix was submitted in toto for histologic examination.

**Figure 1:**
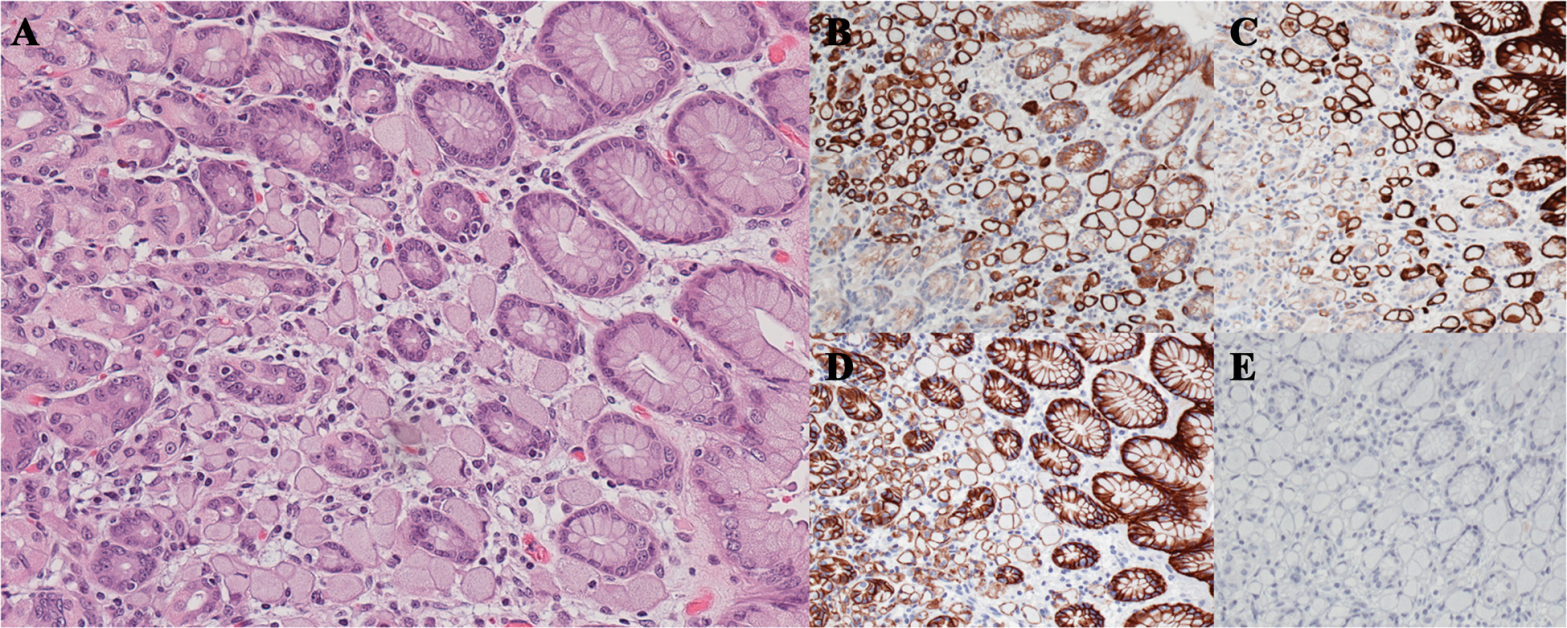
**Intramucosal Gastric Signet Ring Cell Carcinoma (x 20 magnification). (A)** Hematoxylin- and eosin-stained section of gastric mucosa with a focus of infiltrating cells having prominent mucin vacuoles and peripheral displacement of nuclei. By immunohistochemistry, the signet ring cells show strong positivity for Cytokeratin 7 **(B)** and Cytokeratin 20 **(C)**, weak positivity for E-cadherin **(D)**, and negativity for CDX2 **(E)**.

Histology delineated 25 foci of signet ring cell carcinoma confined to the lamina propria (intramucosal) of the proximal gastric fundus. These foci were composed of single cells and loose clusters of cells with mucin vacuoles that dispersed the nucleus to the periphery (Figure 1A); the largest focus was 0.2 cm in diameter. No mural invasion or regional nodal metastases were identified, resulting in a final pathologic stage of pT1N0.

**Figure 2:**
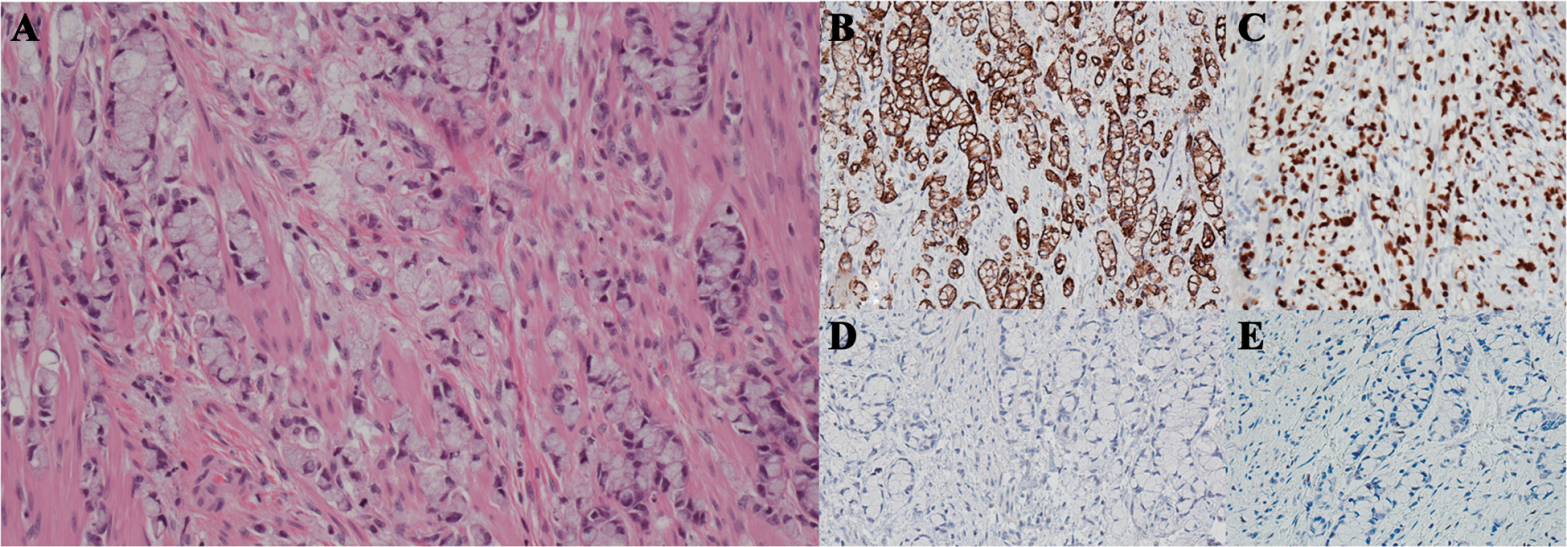
**Appendiceal Signet Ring Cell Carcinoma (x 20 magnification). (A)** Hematoxylin- and eosin-stained section of appendiceal wall with extensive infiltration by columns and groups of cells having prominent mucin vacuoles and peripheral displacement of nuclei. By immunohistochemistry, the signet ring cells show strong positivity for Cytokeratin 20 **(B)** and CDX2 **(C)** and negativity for Cytokeratin 7 **(D)** and estrogen receptor **(E)**.

The appendiceal wall was replaced by an infiltrative neoplasm composed of clusters and columns of cells with mucin vacuoles that dispersed the nucleus to the periphery. Signet ring cells composed more than 50% of the tumor volume, with focal mucin lakes (Figure 2A). The malignancy invaded the lymphatics and the mesoappendiceal adipose tissue. A precursor adenoma with low grade dysplasia replaced the appendiceal mucosa, providing credence for a primary carcinoma. Both the appendiceal signet ring cell carcinoma and adenoma extended to the proximal margin of the appendectomy specimen.

Despite the synchronous gastric and appendiceal carcinomas focally sharing similar histomorphology, they had divergent immunohistochemical phenotypes. The gastric carcinomata were cytokeratin 7 (Figure 1B), cytokeratin 20 (Figure 1C), and weakly E-cadherin positive (Figure 1D), and negative for CDX-2 (Figure 1E), estrogen receptor and mammoglobin. The appendiceal carcinoma was cytokeratin 20 (Figure 2B), CDX-2 (Figure 2C) and E-cadherin positive but negative for cytokeratin 7 (Figure 2D), and estrogen receptor (Figure 2E). In addition, the appendiceal carcinoma was negative for synaptophysin, excluding a neuroendocrine neoplasm.

Given the diagnosis of an appendiceal malignancy, colonoscopy was performed post-operatively and was negative for distal lesions. One week subsequent to the laparotomy, a right hemicolectomy was performed to complete resection and stage the appendiceal tumor. Signet ring cell carcinoma was identified at the appendiceal orifice and had disseminated to three pericolic lymph nodes, with a final pathologic stage of pT3N1. Post-operative recovery was unremarkable; further management included adjuvant chemotherapy.

Pathologic review of the previous mammary carcinoma confirmed an invasive carcinoma of the breast, of lobular phenotype with classic signet ring cell morphology, and associated lobular carcinoma in situ.

## Conclusions

In summary, we present the first case report of an appendiceal signet ring cell carcinoma in an individual with synchronous CDH1-associated hereditary diffuse gastric carcinoma. The occurrence of this unusual tumor, alongside other malignancies with a dyshesive signet ring cell morphology, gives further credence to its inclusion in the spectrum of HDGC disease. With an estimated incidence of 0.12 cases per one million people per year and representing 0.3 to 1.4% of all appendectomy specimens [37-40], primary malignancies of the appendix are a rare occurrence in the general population, and as in this case, are rarely suspected pre-operatively. The majority (38%) of cases in the SEER database are mucinous adenocarcinomas, with signet ring cell carcinomas comprising only 4% [37,38]. The diagnosis of signet ring cell carcinoma is rendered when a tumor contains at least 50% of cells with signet ring cell morphology, often associated with focal mucinous differentiation. A signet ring cell histology in appendiceal tumors is significant as it portends a worse prognosis, with larger tumor sizes at presentation, more advanced stage/distant disease at presentation and worse survival outcomes, as compared to other types of primary appendiceal malignancies [37-39,41].

Prior to making a diagnosis of signet ring cell carcinoma, it is important to exclude a metastasis from a signet ring cell carcinoma at another site; this is critical in the setting of a hereditary cancer syndrome that predisposes to malignancies of similar morphology at different sites. There are reported cases of gastric signet ring cell carcinoma, metastasizing to the appendix, mimicking a primary appendiceal malignancy [42]. Another consideration is that second primary synchronous and metachronous neoplasms occur in up to 35% of patients with an appendiceal carcinoma, typically in the colorectum [43,44]. In our case, although the appendiceal and gastric malignancies focally share similar histologic features, their immunohistochemical patterns are distinct and consistent with a primary malignancy at each site. Furthermore, their immunohistochemical patterns are distinct from the expected staining pattern of a metastatic lobular breast carcinoma, an important consideration given the occurrence of this lesion in the spectrum of HDGC syndrome, and in this patient. In addition, adenomas are a recognized precursor to signet ring cell carcinoma within the colorectum [31] and the association of a villous adenoma with the appendiceal signet ring cell carcinoma, further supports the primary nature of this malignancy. Lastly, no synchronous primary or secondary colorectal neoplasm was identified elsewhere in our patient.

Our patient’s prognosis is adversely affected by the finding of a synchronous signet ring cell carcinoma of the appendix. The ‘prophylactic’ total gastrectomy is established as a curative procedure in HDGC, given the high prevalence rate of early diffuse gastric carcinoma in this cohort. However, this procedure is not without morbidity and should be carefully considered in the management of HDGC, especially since the risk of extra-gastric malignancies is largely unknown. Furthermore, if symptoms attributable to the lower gastrointestinal system exist in these individuals at risk for malignancy at multiple organ sites, consideration should be given for colonoscopy prior to prophylactic gastrectomy to provide more definitive surgical management. It is likely that as more individuals with HDGC syndrome are identified early though genetic screening, and undergo prophylactic gastrectomy, longer survival outcomes will effectively unveil additional risks of malignancy at other organ sites. Further studies are needed to accurately define these extra-gastric malignancies, not only to identify those individuals and families with certain tumor types other than diffuse gastric carcinoma that should be considered for genetic testing, but to also better define the clinical guidelines for the screening of malignancies in individuals with genetically proven HDGC.

## Consent

Written informed consent was obtained from the patient for publication of this Case Report. A copy of the written consent is available for review by the Editor of this journal.

## Abbreviations

HDGC: Hereditary diffuse gastric carcinoma; CDH1: Cadherin-1; E-cadherin.

## Competing interests

The authors declare that they have no competing interests.

## Authors’ contributions

All authors were involved in the acquisition and interpretation of clinico-pathologic data for the case report. LH drafted the manuscript. KJ, NC and SM critically revised the manuscript for important intellectual content. All authors read and approved the final manuscript.

## Pre-publication history

The pre-publication history for this paper can be accessed here:

http://www.biomedcentral.com/1471-230X/13/114/prepub
